# Predictors of home births among rural women in Ghana: analysis of data from the 2014 Ghana Demographic and Health Survey

**DOI:** 10.1186/s12884-020-03211-4

**Published:** 2020-09-10

**Authors:** Eugene Budu

**Affiliations:** Department of Population and Health, University of Cape Coast, Cape Coast, Ghana

**Keywords:** Predictors, Home births, Rural women, Ghana

## Abstract

**Background:**

Home births is one of the factors associated with maternal mortality. This study examined the predictors of home births among rural women in Ghana.

**Methods:**

Data for this study was obtained from the 2014 Demographic and Health Survey (DHS) of Ghana. For the purpose of this study, a sample size of 2,101 women in the rural areas who had given birth within five years prior to the survey and had responses on variables was considered. Data processing, management and analysis were carried out using STATA version 14.0. This study carried out bivariate and multivariate analyses and results were tested at 95% confidence interval. The Adjusted odds ratios were used to present the results and the level of statistical significance was assessed using 95% confidence intervals.

**Results:**

Home births was found to be high among women who resided in the Northern region compared to those in the Western region [AOR, 1.81 CI = 1.10–2.98]. Similarly, the likelihood of home birth was high among women with four or more births [AOR, 1.46 CI = 1.03–2.05] and Traditionalists [AOR, 2.50 CI = 1.54–4.06]. Conversely, giving birth at home was low among women with higher level of education [AOR = 0.58, CI = 0.43–0.78], those with rich wealth status [AOR = 0.19, CI = 0.10–0.38], those with four or more ANC visits [AOR = 0.11, CI = 0.15–0.23] and those who were covered by NHIS [AOR = 0.58, CI = 0.46–0.72].

**Conclusions:**

Over the years, there have been efforts by governments in Ghana to make maternal health services free in the country. However, a substantial proportion of women still undergo home births. To reduce the utilization of home births in Ghana, it is essential that government and non-governmental organisations make the cost of delivery services part of the free maternal health care policy and take into consideration the factors associated with the high rates of home births among rural women in Ghana.

## Background

Labour and birth are essential components of the pregnancy and childbirth continuum as they determine the rates of maternal mortality from pregnancy-related complications and childbirth [1]. Proven health care interventions such as facility births and skill assistance during childbirth can prevent and manage these complications [1]. The provision of skilled attendants in the comprehensive and continuum pattern of care during childbirth has been argued to reduce maternal and neonatal death [2]. In low- and middle-income countries (LMICs), there is high tendency for women in rural areas to give birth at places outside a healthcare facility, where there is no to minimal skilled obstetric care [3, 4]. Most of such births are not done by skilled birth attendants (SBAs) such as doctors, physician assistants, midwives or nurses but rather by traditional birth attendants (TBA), relatives and friends [5].

A recent report by the World Health Organization (WHO) indicates that whereas women in high-income countries have a lifetime risk of dying from pregnancy-related causes is 1 in 3800, that of women in sub-Saharan Africa 1 in 39 [6]. In sub-Saharan Africa, hemorrhage, hypertension, sepsis and obstructed labour that emerge around the time of labour and delivery are the major causes of maternal deaths [7]. To reduce this risk, one of the most effective intervention was the universal skilled birth attendance which in SSA is often equated with facility-based births [6]. Ghana, just like other SSA countries, has this burden of high maternal deaths. Despite a decreases in maternal mortality from 760 to 319 deaths per 100,00 live births between 1990 and 2015, the rate is still high compared to high-income countries [8]. In Ghana, skilled assistance by a doctor, physician assistant, midwife or a nurse during delivery in rural areas is around 45–46.1% [9, 10]. In most instances, women who do not receive skilled attendance during delivery are assisted by either traditional birth attendants (TBAs) or relatives and friends [10]. Lack of skilled birth attendance is a major drawback to achieving SDG 3 target 3.1, which aims at reducing global maternal mortality ratio to less than 70 per 100,000 [11].

Although WHO has recommendations aimed at encouraging health facility births, these numerous challenges impede women in sub-Saharan Africa from assessing skilled assistance during birth. These barriers can be categorised into logistical and social factors. Distance to the facility [12], rural residence [13], lack of health insurance and other economic factors [14, 15] have been found to be associated with lower rates of health facility birth. The social barriers include the social norms about place of delivery, the role of socioeconomic status (SES) and the influence of husband and other family relatives in the selection of a place of delivery [16]. Such barriers also exist in Ghana and is predominant in rural areas, where home births are high compared to urban areas [17, 18]. Despite the high utilization of home births among rural women in Ghana [19], most of the studies on uptake of skilled delivery services including health facility birth have focused on predictors of health facility birth as well as predictors of choice of place of birth among women [17, 18, 20, 21]. To the best of my knowledge, no study has looked at the factors that influence home births among rural women in Ghana. Hence, this study seeks to fill this gap in literature by examining the predictors of home births among rural women in Ghana. Findings from this study will help understand the situation of home births in rural settings and come out with strategies aimed at reducing home births in those settings to help in the reduction of maternal mortality and morbidity cases.

## Methods

### Data source

Data for this study was obtained from the 2014 Demographic and Health Survey (DHS) of Ghana. DHS is a nationwide survey collected every five-year period across LMICs. Women’s files were used for this study which contains the responses of women aged between 15 and 49. Stratified dual-stage sampling approach was employed and the same questions were posed to all women. Selection of clusters (i.e. enumeration areas [EAs]) is the first step in the sampling process followed by systematic household sampling within the selected EAs. And the second step involved the selection of households from the predefined clusters. For the purpose of this study, only women in the rural areas and have given birth 5 years prior to the survey, who had complete cases on all the variables considered for the study were used (*N* = 02,101). Details of the methodology employed by the GDHS can be found in the final report (GSS et al., 2015).

### Study Variables

#### Dependent variable

“Place of delivery” was the dependent variable and was obtained from the question, “Where did you give birth to [NAME]?” Response to this question were coded respondent’s home, other home, government hospital, government health centre/clinic, government health post/CHPS, other public, private hospital/clinic, maternity homes and others. It was then dichotomised to into facility birth = 0 and home birth = 1 where respondent’s home and other home were grouped as “home birth” and all the other categories were grouped as “facility birth” [19, 22, 23].

### Independent variables

The study considered twelve independent variables. These were age, region, religion, ethnicity, educational level, marital status, wealth status, employment, parity, sex of household head, antenatal visits and decision making for healthcare. These variables were not determined a priori; instead, based on parsimony, theoretical relevance and practical significance with place of birth [14, 21, 23, 24].

### Statistical analysis

Data processing, management and analysis were carried out using STATA version 14.0. This study carried out univariate, bivariate and multivariate analyses and results were tested at 95% confidence interval. The univariate analysis was conducted to show the samples (N) and percentage of women across socio-demographic characteristics (Table 1). Bivariate analysis was conducted to show the proportion of home deliveries across socio-demographic characteristics with their significance levels (*p*-values) (Table 1). Multivariate analysis (Binary Logistic Regression) was further conducted. Only the variables that showed statistical significance in the bivariate analysis were used for the regression analysis. The results were presented as crude and adjusted odds ratios with their corresponding 95% confidence intervals signifying their level of precision (Table 2). Statistical significance was declared at *p* < 0.05. Sample weight was applied and the survey command (svy) was applied to cater for the complex sampling design of the survey.

**Table 1:** Home births among women in the rural areas of Ghana

Variable	Weighted N	Weighted %	Proportion of women who gave birth at home	*p*-value
**Age**				*p* = 0.151
15–24	402	19.1	17.1	
25–34	981	46.7	47.4	
35–49	718	34.2	35.5	
**Region**				*p* < 0.001
Western	241	11.5	9.0	
Central	281	13.3	8.8	
Greater Accra	77	3.7	1.5	
Volta	189	9.0	8.7	
Eastern	212	10.1	10.2	
Ashanti	286	13.6	3.8	
Brong Ahafo	190	9.1	8.6	
Northern	390	18.5	32.2	
Upper East	136	6.5	4.8	
Upper West	100	4.8	12.3	
**Occupation**				*p* = 0.789
Working	314	14.9	14.0	
Not working	1787	85.1	86.0	
**Ethnicity**				*p* < 0.001
Akan	872	41.5	25.5	
Ga/Dangme	83	4.0	3.3	
Ewe	261	12.4	10.2	
Guan	45	2.1	2.2	
Mole Dagbani	462	22.0	28.0	
Grussi	69	3.3	4.8	
Gruma	278	13.2	25.3	
Mande	31	1.5	0.7	
**Education**				*p* < 0.001
No education	813	38.7	58.6	
Primary	456	21.7	21.3	
Higher	832	39.6	20.1	
**Wealth**				*p* < 0.001
Poor	1423	67.7	86.7	
Middle	425	20.2	12.0	
Rich	254	12.1	1.3	
**Parity**				*p* < 0.001
One birth	303	14.4	9.3	
Two births	340	16.2	14.2	
Three births	389	18.5	16.0	
Four or more births	1069	50.9	60.5	
**Religion**				p < 0.001
Christianity	1541	72.9	63.0	
Islam	367	17.4	17.9	
Traditionalist	105	4.9	10.4	
No religion	102	4.8	8.8	
**Marital status**				*p* = 0.094
Married	1529	72.7	74.3	
Cohabiting	572	27.3	25.7	
**Sex of household head**				*p* = 0.125
Male	1775	84.5	87.8	
Female	323	15.5	12.2	
**ANC visits**				*p* < 0.001
No ANC visits	80	3.8	9.4	
1–3 visits	250	11.9	20.8	
4 or more visits	1771	84.3	69.8	
**Healthcare decision making**				*p* = 0.441
Not alone	1636	77.9	81.2	
Respondent alone	465	22.1	18.8	
**Frequency of reading newspaper/magazine**				
Not at all	1969	93.7	97.7	*p* < 0.001
Less than once a week	70	3.5	1.2	
At least once a week	62	2.9	1.1	
**Frequency of listening to radio**				*p* < 0.001
Not at all	434	20.7	29.7	
Less than once a week	703	33.4	32.0	
At least once a week	964	45.9	38.3	
**Frequency of watching television**				*p* < 0.001
Not at all	948	45.1	63.0	
Less than once a week	518	24.6	17.4	
At least once a week	635	30.3	19.6	
**Covered by NHIS**				*p* > 0.001
No	668	32.5	40.1	
Yes	1419	67.5	59.9	

Source: Computed from 2014 GDHS

**Table 2:** Logistic regression analysis on predictors of home births among women in the rural areas of Ghana

Variable	COR	AOR
**Region**		
Western	1	1
Central	1.03 (0.69–1.53)	0.89 (0.55–1.42)
Greater Accra	0.70 (0.35–1.41)	0.89 (0.34–2.35)
Volta	1.25 (0.83–1.89)	0.76 (0.42–1.37)
Eastern	1.47 (0.99–2.20)	1.02 (0.63–1.34)
Ashanti	0.55^*^ (0.34–0.90)	0.51^*^ (0.30–0.87)
Brong Ahafo	0.81 (0.55–1.20)	0.61^*^ (0.40–0.93)
Northern	3.96^***^ (2.79–5.63)	1.81^*^ (1.10–2.98)
Upper East	0.37^***^ (0.24–0.57)	0.22^***^ (0.13–0.38)
Upper West	1.21 (0.83–1.75)	0.77 (0.47–1.27)
**Ethnicity**		
Akan	1	1
Ga/Dangme	1.47 (0.88–2.44)	1.01 (0.51–1.99)
Ewe	1.36 (0.99–1.87)	1.10 (0.67–1.82)
Guan	1.17 (0.65–2.10)	0.56 (0.27–1.17)
Mole Dagbani	1.22 (0.97–2.10)	1.03 (0.67–1.57)
Grussi	1.39 (0.91–2.12)	1.39 (0.78–2.47)
Gruma	5.01^***^ (3.73–6.72)	1.14 (0.69–1.89)
Mande	0.47 (0.19–1.45)	0.50 (0.17–1.47)
**Education**		
No education	1	1
Primary	0.58^***^ (0.46–0.73)	0.86 (0.64–1.14)
Higher	0.31^***^ (0.25–0.38)	0.58^***^ (0.43–0.78)
**Wealth**		
Poor	1	1
Middle	0.54^***^ (0.42–0.69)	0.79 (0.57–1.08)
Rich	0.11^***^ (0.06–0.20)	0.19^***^ (0.10–0.38)
**Parity**		
One birth	1	1
Two births	1.38 (0.98–1.94)	1.27 (0.85–1.88)
Three births	1.47^*^ (1.05–2.05)	1.33 (0.90–1.97)
Four or more births	2.20^***^ (1.65–2.93)	1.46^*^ (1.03–2.05)
**Religion**		
Christianity	1	1
Islam	1.20 (0.95–1.52)	0.97 (0.71–1.33)
Traditionalist	4.92^***^ (3.24–7.47)	2.50^***^ (1.54–4.06)
No religion	3.94^***^ (2.59–5.99)	2.39^***^ (1.53–3.74)
**ANC visits**		
No ANC visits	1	1
1–3 visits	0.19^***^ (0.09–0.42)	0.24^***^ (0.11–0.55)
4 or more visit	0.05^***^ (0.02–0.11)	0.11^***^ (0.15–0.23)
**Frequency of reading newspaper**		
Not at all	1	1
Less than once a week	0.32^**^ (0.16–0.63)	0.90 (0.42–1.93)
At least once a week	0.49 (0.23–1.05)	1.71 (0.72–4.07)
**Frequency of listening to radio**		
Not at all	1	1
Less than once a week	0.61^***^ (0.48–0.78)	1.01 (0.75–1.35)
At least once a week	0.41^***^ (0.33–0.52)	0.84 (0.64–1.10)
**Frequency of watching television**		
Not at all	1	1
Less than once a week	0.62^***^ (0.49–0.78)	1.07 (0.80–1.44)
At least once a week	0.51^***^ (0.41–0.63)	0.95 (0.73–1.25)
**Covered by NHIS**		
No	1	1
Yes	0.51^***^ (0.42–0.61)	0.58^**^ (0.46–0.72)
N	2,101	2,101
Pseudo R^2^		0.187

Exponentiated coefficients; 95% confidence intervals in brackets,^*^p < 0.05,^**^p < 0.01,^***^p < 0.001, *cOR *crude Odds Ratios, *aOR *adjusted Odds Ratios

### Ethics approval and consent to participate

The survey reported that ethical approval was granted by the Institutional Review Board of ICF International and Ethical Review Committee of Ghana Health Service [19]. I further obtained permission from the DHS Program for use of this data for the study. The data can be accessed from their website (www.measuredhs.com ).

## Results

Table 1 presents results on the prevalence of home births among women in the rural areas of Ghana by their socio-demographic characteristics. The results show that home births were high among women aged 25–34(47.4%), those in the Northern region (32.2%), non-working women (86.0%), those of the Mole Dagbani Ethnic group (28.0%), those with poor wealth status (86.7%), those with parity four or more (60.5%), Christians (63.0%) and those with no education (58.6%). Home births were also high among women with male household heads (87.8%), those who had four or more ANC visits (69.8%), those who did not take healthcare decisions alone (84.2%), those who neither read newspaper/magazine (97.7%) nor watched television (63.0%), those who listened to radio at least once a week (38.3%) and those who were covered by NHIS (59.9%). The results further showed significant associations between home births and all the explanatory variables except age, occupation, marital status, sex of household head and healthcare decision-making (see Table 1).

### Multivariable regression analysis results

Table 2 shows results of the binary logistic regression analysis on the factors that influence home births among women in the rural areas of Ghana. The results show that women in the Northern region were more likely to give birth at home compared to those in the Western region [AOR, 1.81 CI = 1.10–2.98]. Similarly, the likelihood of home birth was high among women with four or more births [AOR, 1.46 CI = 1.03–2.05] and Traditionalists [AOR, 2.50 CI = 1.54–4.06]. Conversely, home births was low among women with higher level of education [AOR = 0.58, CI = 0.43–0.78], those with rich wealth status [AOR = 0.19, CI = 0.10–0.38], those who had four or more ANC visits [AOR = 0.11, CI = 0.15–0.23] and those who were covered by NHIS [AOR = 0.58, CI = 0.46–0.72].

## Discussion

Home births in Sub-Saharan Africa, is one of the risk factors for maternal mortality [25]. This study examined the predictors of home births among rural women in Ghana. Home births was found to be high among women who lived in the Northern region of Ghana. Similar findings were obtained by Gudu and Addo [20] and Adatara et al. [26] in their studies on factors associated with utilization of skilled service births among women in rural northern Ghana and cultural beliefs and practices of women influencing home births in rural Northern Ghana, respectively. These authors provided several reasons for the high utilization of home births in the region, including cultural practices and low socio-economic status. The cultural practices, which are more predominant among women who are traditionalists, support the findings that the likelihood of home birth is high among women who belonged to the Traditional religion. Another possible reason for this finding is that although giving birth at health facility is free in Ghana, costs such as supplies and medications, transportation, unofficial provider fees may deter women who live in low-income settings. These are costs not covered under the current user-fee exemption policy and hence pregnant women may rather opt for TBAs, whose services are often cheap and considered negotiable [27].

In line with this, this study identified that the likelihood of home birth decreased among rural women with high wealth status and educational level as well as those covered by NHIS. Lack of health insurance and low socio-economic status have been found in previous studies as barriers to health facility births [14–16]. Hence, rural women who are unable to pay for cost of transportation to health facilities as well as the cost of services offered during health facility births may have no alternative than to give birth at home. A recent study on home births among rural women in northern Ghana explained that women choose to give birth at home because they are discouraged by the expensive supplies, including sanitary pads, disinfectants, napkins, and mackintosh that they are obliged to bring along for giving birth at the health facility. Their inability of to purchase these items due to low-income status and the attendant humiliation by the midwives hinders most of them from seeking health facility births [28]. Women with some level of education are also less likely to give birth at home, compared to those with no level of education probably due inadequate access to information on the risks associated with home births [25, 29].

The likelihood of home births was high among women with four or more births but was low among those who had four or more ANC visits. Available literature supports the association between higher parity and low utilization of skilled birth attendance during births [30]. Conversely, use of health facility during births has been characterized by first and low-order births [31]. Parity has been found to be strongly associated with health seeking behavior, with a number of studies indicating that women with first pregnancies are more inclined to health facility than any other parity group [32–34]. Women with high parity are often regarded as having the confidence through their cumulative birth giving experiences to opt for an unskilled birth attendance. [35]. Four or more ANC visits decreased the likelihood of home births in the current study because a woman with at least four antenatal visits, has a greater chance of obtaining adequate information on birth preparedness and this decreases the likelihood of home births [36, 37].

### Strengths and limitation

The strength of the study lies in the relatively large sample size that gave the study the statistical power to run rigorous analysis. The sampling employed also makes the data collected nationally representative and for that matter findings from such a study can also be generalisable to similar populations in Ghana. However, some potential limitations inherent within the study are captured. First, the use of the DHS large dataset for analysis shams the ability to establish causality but only association. Second, there is also the possibility of social desirable inherent with self-reporting and recall bias from the respondents. Other limitations could be errors associated with data extraction and curation and coding of procedures in charts and tables [38]. However, the coding and data extraction did not go through substantial changes.

## Conclusions

Over the years, there have been efforts by governments in Ghana to offer free maternal health services in the county. However, a significant number of women still give birth at home. This study has shown that women’s region of residence, educational status, wealth quintile, parity, religion, number of ANC visits and if the respondent is covered by NHIS have significant influence on women’s use of home birth services. To reduce the utilization of home births in Ghana, there is the need for government and non-governmental organisations to integrate the cost of birth services into the free maternal health care policy by encouraging more ANC visits, economic empowerment of women, encouraging female, girl child education, strengthening the NHIS to cover some of the hidden cost and education in general on the need to deliver at health facilities especially among those who are traditionalist since they might hold certain cultural believes.

## Data Availability

All the data for this study are openly available at https://dhsprogram.com/data/available-datasets.cfm.

## Abbreviations

GDHS: Ghana Demographic and Health Survey
TBA: Traditional Birth Attendant
LMICs: Low and Middle Income Countries
WHO: World Health Organization
SSA: Sub-Saharan Africa
